# CCR1 Plays a Critical Role in Modulating Pain through Hematopoietic and Non-Hematopoietic Cells

**DOI:** 10.1371/journal.pone.0105883

**Published:** 2014-08-29

**Authors:** Nuruddeen D. Lewis, Akalushi Muthukumarana, Steven E. Fogal, Laura Corradini, Dimitria E. Stefanopoulos, Prathima Adusumalli, Josephine Pelletier, Mark Panzenbeck, Karen Berg, Melissa Canfield, Brian N. Cook, Hossein Razavi, Daniel Kuzmich, Shawn Anderson, Devan Allard, Paul Harrison, Christine Grimaldi, Donald Souza, Christian Harcken, Ryan M. Fryer, Louise K. Modis, Maryanne L. Brown

**Affiliations:** 1 Department of Immunology & Inflammation, Boehringer Ingelheim Pharmaceuticals, Inc., Ridgefield, Connecticut, United States of America; 2 Department of Cardiometabolic Diseases Research, Boehringer Ingelheim Pharmaceuticals, Inc., Ridgefield, Connecticut, United States of America; 3 Department of Medicinal Chemistry, Boehringer Ingelheim Pharmaceuticals, Inc., Ridgefield, Connecticut, United States of America; 4 Department of Integrative Toxicology, Boehringer Ingelheim Pharmaceuticals, Inc., Ridgefield, Connecticut, United States of America; 5 Department of CNS Diseases Research, Boehringer Ingelheim Pharma GmbH & Co KG, Biberach, Germany; Boston University, United States of America

## Abstract

Inflammation is associated with immune cells infiltrating into the inflammatory site and pain. CC chemokine receptor 1 (CCR1) mediates trafficking of leukocytes to sites of inflammation. However, the contribution of CCR1 to pain is incompletely understood. Here we report an unexpected discovery that CCR1-mediated trafficking of neutrophils and CCR1 activity on non-hematopoietic cells both modulate pain. Using a genetic approach (CCR1^−/−^ animals) and pharmacological inhibition of CCR1 with selective inhibitors, we show significant reductions in pain responses using the acetic acid-induced writhing and complete Freund's adjuvant-induced mechanical hyperalgesia models. Reductions in writhing correlated with reduced trafficking of myeloid cells into the peritoneal cavity. We show that CCR1 is highly expressed on circulating neutrophils and their depletion decreases acetic acid-induced writhing. However, administration of neutrophils into the peritoneal cavity did not enhance acetic acid-induced writhing in wild-type (WT) or CCR1^−/−^ mice. Additionally, selective knockout of CCR1 in either the hematopoietic or non-hematopoietic compartments also reduced writhing. Together these data suggest that CCR1 functions to significantly modulate pain by controlling neutrophil trafficking to the inflammatory site and having an unexpected role on non-hematopoietic cells. As inflammatory diseases are often accompanied with infiltrating immune cells at the inflammatory site and pain, CCR1 antagonism may provide a dual benefit by restricting leukocyte trafficking and reducing pain.

## Introduction

CC chemokine receptor 1 (CCR1) is a G-protein coupled receptor that mediates trafficking of leukocytes to sites of inflammation [1] and is a therapeutic target for the treatment of inflammatory diseases. CCR1 has several known ligands including MIP-1α/CCL3, RANTES/CCL5, and MCP3/CCL7 [2]. In humans, CCR1 is highly expressed on monocytes, whereas in rodents, it is primarily expressed on neutrophils [1], [3]. Due to its role in leukocyte trafficking, mice lacking CCR1 develop milder forms of disease in several pre-clinical mouse models of inflammatory diseases including collagen-induced arthritis [4] and experimental autoimmune encephalomyelitis [5].

Inflammatory diseases are associated with both increased leukocyte infiltration into the inflammatory site and pain [6]. The relationship between these two processes, however, is not understood, and many questions remain as to how these processes are interconnected [7]. Inflammatory cells have been shown to promote pain through a variety of mechanisms, such as the production of proinflammatory cytokines and chemokines [7]. In addition to their chemotactic role on leukocytes, cytokines and chemokines may act directly on sensory neurons, leading to sensitization and hyperalgesia [8]. Cytokines may also influence pain indirectly by stimulating the release of other inflammatory mediators such as prostaglandins [9].

Due to the strong link between inflammation and pain, we aimed to test whether CCR1 contributes to the induction of pain. To test this, we generated CCR1^−/−^ mice and two novel CCR1 antagonists and evaluated the function of CCR1 in pre-clinical rodent models of inflammation and pain. Consistent with previously published reports, we demonstrate that CCR1 deletion or antagonism with a small molecule restricts immune cell trafficking in a peritonitis model and reduces disease severity in a model of collagen antibody-induced arthritis (CAIA). However, we also demonstrate that CCR1 deletion or antagonism significantly reduces acetic acid-induced writhing and complete Freund's adjuvant (CFA)-induced mechanical hyperalgesia. Reductions in acetic acid-induced writhing coincided with decreased numbers of myeloid cells in the peritoneal cavity. We show that CCR1 is highly expressed on circulating neutrophils and that depletion of neutrophils reduced the writhing response. We further demonstrate using bone marrow transplants that CCR1 activity on both hematopoietic and non-hematopoietic cells is necessary to generate a complete writhing response. Our results suggest that CCR1 modulates pain through two independent mechanisms - neutrophil trafficking to the inflammatory site and through a role on non-hematopoietic cells.

## Methods

### Reagents

CCR1^−/−^ mice were generated by Artemis Pharmaceuticals GmbH (now Taconic Farms) using targeted deletion of exon 2 causing a removal of the open reading frame. Knockout mice were confirmed by Taqman PCR using the following primers for CCR1: Forward- CCAGAGCATTTATGGAGACAACAGT; Reverse- CATCCCAGCTCTGAAATGATAGGA; Probe- CTCTTCTGCCTCTAATCAC. CCR1 inhibitors from the azaindazole class were generated as described [10] and the off-target selectivity profile was assessed in a selectivity screen at a standard concentration of 10 µM and tested in duplicate (Eurofins Panlabs, Taipei, Taiwan) as described [11]. The methods specific to each assay performed can be found at www.eurofinspanlabs.com/Panlabs using the assay number listed in parentheses after each assay: Adenosine A1 (200510), Adenosine A2A (200610), Adrenergic α1A (203100), Adrenergic α1B (203200), Adrenergic β1 (204010), Adrenergic β2 (204110), Calcium Channel L-Type, Dihydropyridine site (214600), Cannabinoid CB1 (217030), Dopamine D1 (219500), Dopamine D2S (219700), GABAA, Flunitrazepam, central (226600), GABAA, Muscimol, Central (226500), Glutamate, NMDA, Phencyclidine (233000), Histamine H1 (239610), Imidazoline I2, Central (241000), Muscarinic M2 (252710), Muscarinic M3 (252810), Nicotinic Acetylcholine (258590), Nicotinic Acetylcholine α1, Bungarotoxin (258700), Norepinephrine Transporter (NET; 204410), Opiate μ (260410), Phorbol Ester (264500), Potassium Channel, KATP (265600), Potassium Channel, hERG (265900), Prostanoid EP4 (268420), Rolipram (270000), Serotonin 5-HT2B (271700), Sigma σ1 (278110), Sodium Channel, Site 2 (279510), Androgen (testosterone) AR (285010), Estrogen ERα (226010), Estrogen ERβ (226050), Progesterone (268000), Retinoid X Receptor RXRα (327147), Thyroid Hormone (285900), Vitamin D3 (288010), Farnesoid X Receptor (311600), Liver X Receptor alpha (331810), Liver X Receptor beta (331820), Peroxisome Proliferator Activated Receptor alpha (338200), Peroxisome Proliferator Activated Receptor gamma (338250), and Retinoic Acid Receptor alpha (338600). The corresponding author should be contacted for all reagent requests.

### Receptor internalization

All animal studies were performed under protocols approved by the Institutional Animal Care and Use Committee at Boehringer Ingelheim Pharmaceuticals and in accordance with the United States Animal Welfare Act. Blood was isolated from C57BL/6 mice and treated with the CCR1 inhibitors at various concentrations at 37°C for 30 minutes. MIP-1α-Alexa-647 (Almac Group) was added to the samples at a 10 nM concentration. Samples were incubated at 37°C for 40 minutes. The reaction was stopped by placing the plate on ice. Red blood cells were then lysed and the samples were washed in PBS. Samples were then washed with an acid wash buffer (0.2 M acetic acid and 0.5 M sodium chloride) to remove surface bound staining. Cells were then stained with anti-mouse Gr-1/Ly6G (R&D Systems) and analyzed by flow cytometry.

### Thioglycollate-induced peritonitis

Mice were dosed orally with BI33 1 hour before being injected intraperitoneally with 3% Brewer Modified BBL Thioglycollate Medium (BD). After 4 hours, cells were isolated by injecting 7 ml of Hank's balanced salt solution containing 2% FBS and 1 mM EDTA. Cells were then counted.

### Collagen antibody-induced arthritis

B10.RIII-*H2^r^ H2-T18^b^*/(71NS)SnJ (The Jackson Laboratory) received an intraperitoneal injection of 2 mg of ArthritoMab antibody cocktail (MD Biosciences) on day 0. On day 3, 37.5 µg of LPS was administered intraperitoneally. BI33 was administered orally each day beginning on Day 0. Beginning on Day 3, mice were checked daily and scored for the onset and development of arthritis. Arthritic severity was recorded using a visual scoring system. The scoring system is 0 = normal; 1 = erythema and edema in 1–2 digits; 2 =  erythema and edema in >2 digits or mild erythema and edema, usually in the ankle joint; 3 = moderate erythema and edema encompassing the tarsal joint; 4 = severe erythema and edema encompassing the tarsal and metatarsal joint.

### Acetic acid-induced writhing

CCR1 inhibitors were administered to C57BL/6 mice by oral gavage at the indicated concentrations. Mice were then tested in the acetic acid-induced writhing model. Mice received an intraperitoneal injection of 10 ml/kg of 0.625% acetic acid. Thirty minutes after the injection, mice were then monitored for an additional 30 minutes and the number of writhes was counted. The cumulative number of writhes during the 30 minute period is shown. Experimenters were blinded to assessment.

### CFA-induced mechanical hyperalgesia

CFA suspension (25µg in 50 µl, Sigma-Aldrich) was injected into the left hind paw of male Han-Wistar rats (Charles River Laboratories, Germany). At 24 hours after CFA administration, BI64 was orally dosed at 30 mg/kg and mechanical hypersensitivity assessed at 2 hrs post-dosing by the paw pressure test (Randall-Selitto, Ugo Basile Equipment) using a cutoff value of 500 g. A vehicle treated group (n = 10) was used as a negative control and indomethacin (30 mg/kg) was used as positive control. Analgesic activity was evaluated by comparing the paw withdrawal threshold of the inflamed paw in the vehicle and compound-treated groups. These experimental procedures were approved by the Ethics committee and the Regierungspräsidium Tübingen and adhered to the guidelines of the Committee for Research and Ethical Issues of IASP 1983.

### Isolation and analysis of cells by flow cytometry

Peritoneal cells were isolated by injecting intraperitoneally 10 ml of Hank's balanced salt solution containing 2% FBS and 1 mM EDTA. The peritoneum was massaged and the fluid was collected and stored on ice. Cells were washed and resuspended in Cell Staining Buffer (BioLegend) containing 10 µg/ml of TruStain fcx (BioLegend) and incubated on ice for 10–15 min. Cells were stained with antibodies against CD11b (M1/70), F4/80 (BM8), B220 (RA3-6B2), Ly6G (1A8), CD45.1 (A20), CD45.2 (104), and CCR1 (643854). All antibodies were purchased from BioLegend except anti-CCR1 which was purchased from R&D Systems. Whole blood was isolated and stained as described. Cells were then washed twice and analyzed on the BD FACS Canto II (BD Biosciences).

### Injection of WT leukocytes

WT leukocytes were obtained by injecting thioglycollate intraperitoneally into WT mice. After 4 hours, peritoneal cells were isolated, washed, counted, and analyzed for viability. Mice were injected intraperitoneally with 2.9 million cells and the mice were immediately tested in the acetic acid-induced writhing model. A portion of the cells was also analyzed by flow cytometry to determine the cellular composition.

### Depletion of neutrophils

Neutrophil depletion was performed by injecting mice intraperitoneally with 500 µg of Ultra-LEAF purified anti-Ly6G antibody (1A8; BioLegend). Ultra-LEAF purified Rat anti-IgG2a antibody was used as a control. Twenty-four hours after dosing, mice were used in the acetic acid-induced writhing model. After the writhing experiment, neutrophils in the blood and peritoneal cavity were assessed by flow cytometry to determine the level of depletion. We confirmed our population was indeed neutrophils by gating on CD11b-positive, side-scatter high, F4/80-negative cells. We compared this gating strategy to the Ly6G-positive population in the isotype control treatment group and found similar results.

### Bone marrow transplants

B6.SJL-*Ptprc^a^ Pepc^b^*/BoyJ (Pep Boy, CD45.2) and WT C57BL/6 mice were purchased from The Jackson Laboratory. Mice were irradiated twice with 500 rad within six hours. The following day, mice received 10–20 million bone marrow cells by intravenous injection. Mice were treated with Baytril in their drinking water for two weeks. After 10 weeks, blood was isolated and profiled for engraftment by analyzing CD45.1 and CD45.2 expression. Only mice with confirmed engraftment over 75% were included in these studies.

## Results

### CCR1 is important for leukocyte trafficking and the severity of arthritis

**Figure 1:**
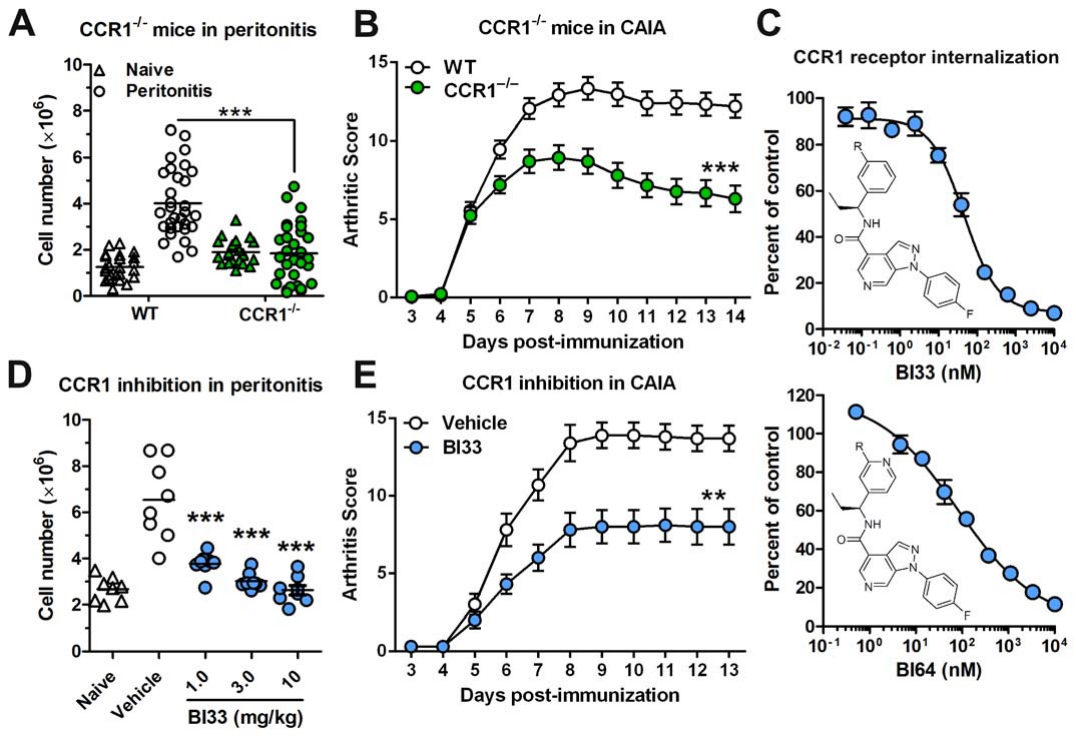
Knockout or inhibition of CCR1 decreases peritoneal inflammation and CAIA. (*A*) Peritoneal cell counts were assessed in WT and CCR1^−/−^ mice in both naïve mice (triangles) and mice that underwent the 4 hr peritonitis (circles) model (*n* = 20 for naïve and 30 for peritonitis). (*B*) Mean arthritic scores of CAIA in WT and CCR1^−/−^ mice (*n* = 22 for WT and 30 for CCR1^−/−^). (*C*) CCR1 receptor internalization on mouse neutrophils was assessed using two CCR1 inhibitors, BI33 and BI64 (*n* = 3). The structure of the CCR1 inhibitors is also shown along with the IC_50_ values. (*D*) Peritoneal cell counts were assessed in mice in a 4 hr model of thioglycollate-induced peritonitis after being dosed with a CCR1 inhibitor or a vehicle (*n* = 8). Cell counts from naïve mice (triangles) in the absence of the inhibitor are also shown (*n* = 8) (*E*) Mean arthritic scores of CAIA in mice are shown (*n* = 10). Mice were dosed twice daily with a CCR1 inhibitor or a vehicle. **P<0.01, and ***P<0.001.

In order to explore the role of CCR1 in inflammation and pain, we generated CCR1^−/−^ mice and confirmed a reduction in immune cell trafficking utilizing a 4 hr thioglycollate-induced peritonitis model. As expected, deletion of CCR1 caused a significant reduction in leukocyte trafficking to the peritoneum (Fig. 1A). Similarly, we tested the CCR1^−/−^ mice in the pre-clinical CAIA mouse model, which is characterized by immune cell trafficking into the joints. Mice deficient in CCR1 displayed a significant reduction in joint inflammation and the severity of disease as shown by the arthritis score (Fig. 1B).

In parallel, we evaluated the effects of two novel, potent CCR1 inhibitors, BI33 and BI64. Both inhibitors blocked CCR1 internalization on mouse neutrophils in whole blood with IC_50_ values of 46 nM for BI33 and 80 nM for BI64 (Fig. 1C). The selectivity of these compounds was confirmed using the HitProfilerScreen (Eurofins Panlabs) as described in the methods (data not shown). We tested their potency in vivo using both the peritonitis and CAIA mouse models of inflammation. In acute peritonitis, BI33 at 1, 3, and 10 mg/kg doses resulted in significantly fewer cells in the peritoneum compared to vehicle controls (Fig. 1D). In CAIA, the arthritis score of mice treated with BI33 at 30 mg/kg was also significantly reduced to a level comparable with CCR1-deficient mice (Fig. 1E). These data confirm the utility of our CCR1^−/−^ mice and CCR1 antagonists as tools in evaluating the role of CCR1 in vivo.

### Deletion or inhibition of CCR1 decreases pain responses

**Figure 2:**
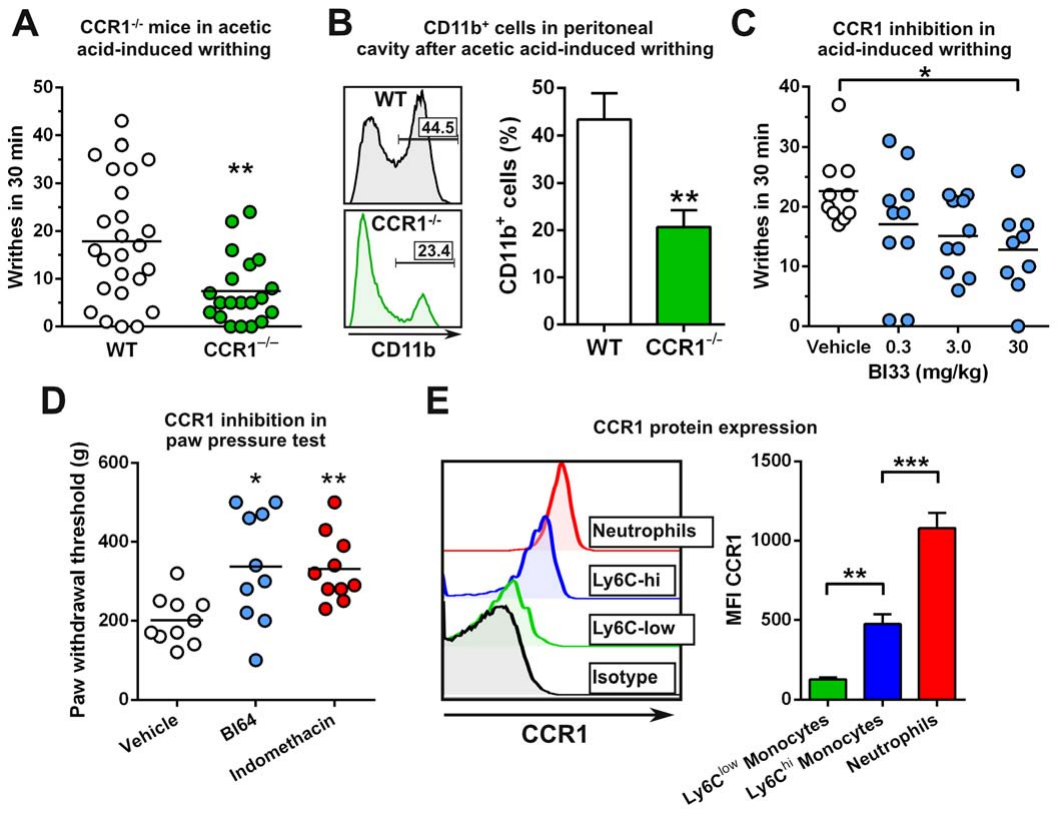
Knockout or inhibition of CCR1 decreases writhing which coincides with reduced myeloid cell recruitment. (*A*) C57BL/6 WT and CCR1^−/−^ mice were assessed in the acetic acid-induced writhing model. The number of writhes counted within 30 minutes is shown for each mouse (*n* = 12 for WT and 9 for CCR1^−/−^). (*B*) Peritoneal cells were assessed after the acetic acid-induced writhing test and the percentage of CD11b^+^ cells is shown (*n* = 5). (*C*) C57BL/6 mice were treated with BI33 or vehicle. After 30 min, mice were assessed in the acetic acid-induced writhing model (*n* = 9 or 10). (*D*) Han-Wistar rats were injected with CFA in the hind paw. Twenty-four hours later, rats were dosed with vehicle, BI64, or indomethacin at 30 mg/kg and assessed for mechanical hypersensitivity 2 hours later in the Randall-Selitto paw pressure test. The paw withdrawal threshold is shown in grams (*n* = 10). (*E*) CCR1 protein expression was assessed on blood CD11b^+^ cells (*n* = 6). *P<0.05, **P<0.01, and ***P<0.001.

Given the crucial role of CCR1 in mediating cellular infiltration to inflammatory sites and the close relationship between inflammation and pain, we wanted to determine if CCR1 regulates the induction of pain. To achieve this, we tested the CCR1^−/−^ mice in the acetic acid-induced writhing model of visceral nociception and found that they had a 58% reduction in the number of writhes (a pain-elicited behavior) as compared to WT mice (Fig. 2A). Analysis of cellular infiltrates in WT mice revealed the presence of infiltrating myeloid (CD11b^+^) cells in the peritoneal cavity after acetic-acid administration. However, the percentage of infiltrating CD11b^+^ cells was significantly reduced by 52% in CCR1-deficient mice, which coincided with the reduction observed in writhing (Fig. 2B). Additionally, we tested the ability of anti-CCL3 and anti-CCL5 antibodies to reduce the writhing response and found that mice that received the anti-CCL3 and anti-CCL5 antibodies writhed less than those that received the isotype control antibody (Fig. S1). However, the total number of writhes in the isotype control antibody-treated group was less than what was normally observed without treatment, suggesting that the injection of IgG alone can decrease the writhing response.

To corroborate our findings and test whether pharmacological inhibition of CCR1 affects pain, we took two approaches. First, we tested BI33 in the acetic acid-induced writhing model and found that it produced dose-dependent decreases in the cumulative number of writhes measured after acetic acid injection, with significance being achieved at the highest tested dose of 30 mg/kg (Fig. 2C). The variability in this model may have contributed to the lack of significance at the lower doses. However, these data are comparable to our observations using CCR1-deficient mice. Second, we performed the Randall-Selitto paw pressure test in a model of CFA-induced mechanical hyperalgesia. Treatment with BI64 at 30 mg/kg resulted in a significant increase (68%) in the paw withdrawal threshold compared to the vehicle-treated group (Fig. 2D). This increase in the withdrawal threshold was comparable to indomethacin, a well-characterized inhibitor of pain (Fig. 2D). Taken together, our data suggest that CCR1 plays a critical role in modulating pain.

### Neutrophil depletion reduces acetic acid-induced writhing

**Figure 3:**
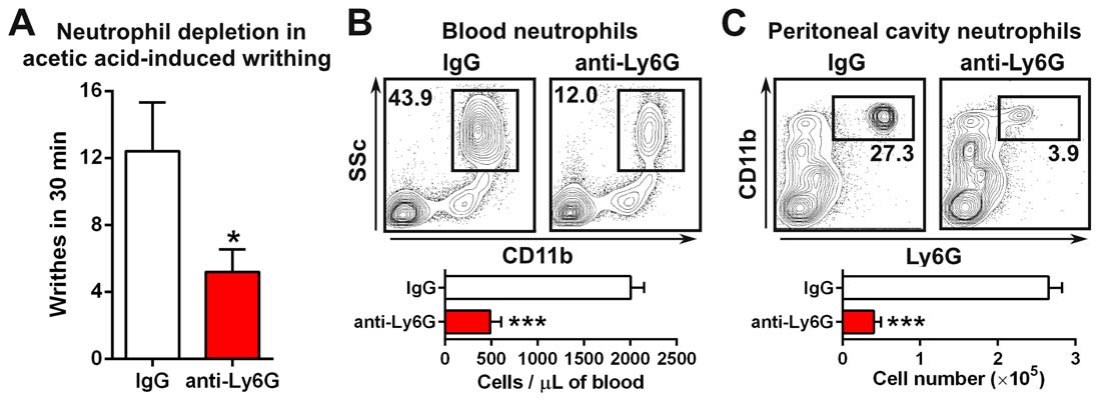
Depletion of neutrophils reduces writhing. (*A*) C57BL/6 mice were treated with anti-Ly6G antibodies or isotype controls for 24 hours and tested in the acetic acid-induced writhing model (*n* = 5). Reduced numbers of neutrophils was confirmed in the blood (*B*) and the peritoneal cavity (*C*) after the writhing model (*n* = 5). *P<0.05 and ***P<0.001.

As the reduction in writhes correlated with reductions in myeloid cells infiltrating the peritoneum, we wanted to test whether depletion of neutrophils would impact the writhing response. We confirmed that neutrophils (CD11b^+^, side-scatter^high^, F4/80^−^) expressed high levels of CCR1 and Ly6C^high^ monocytes expressed moderate levels of CCR1 (Fig. 2E). Neutrophils accumulate at inflammatory sites and have been implicated to modulate pain [11]. We found that treatment with the anti-Ly6G antibody to deplete neutrophils [12] resulted in a 58% relative reduction in writhes as compared to the isotype control treatment group (Fig. 3A). To test the effectiveness of the depletion, we analyzed the number of neutrophils in the blood and peritoneal cavity after the writhing experiment. We observed a 76% reduction in neutrophils in the blood (Fig. 3B) and an 85% reduction in the peritoneal cavity (Fig. 3C). These data demonstrate that neutrophils express high levels of CCR1, traffic to the site of pain, and suggest that they can modulate the acetic acid-induced writhing response.

### Signaling of CCR1 on hematopoietic and non-hematopoietic cells contributes to the writhing response

**Figure 4:**
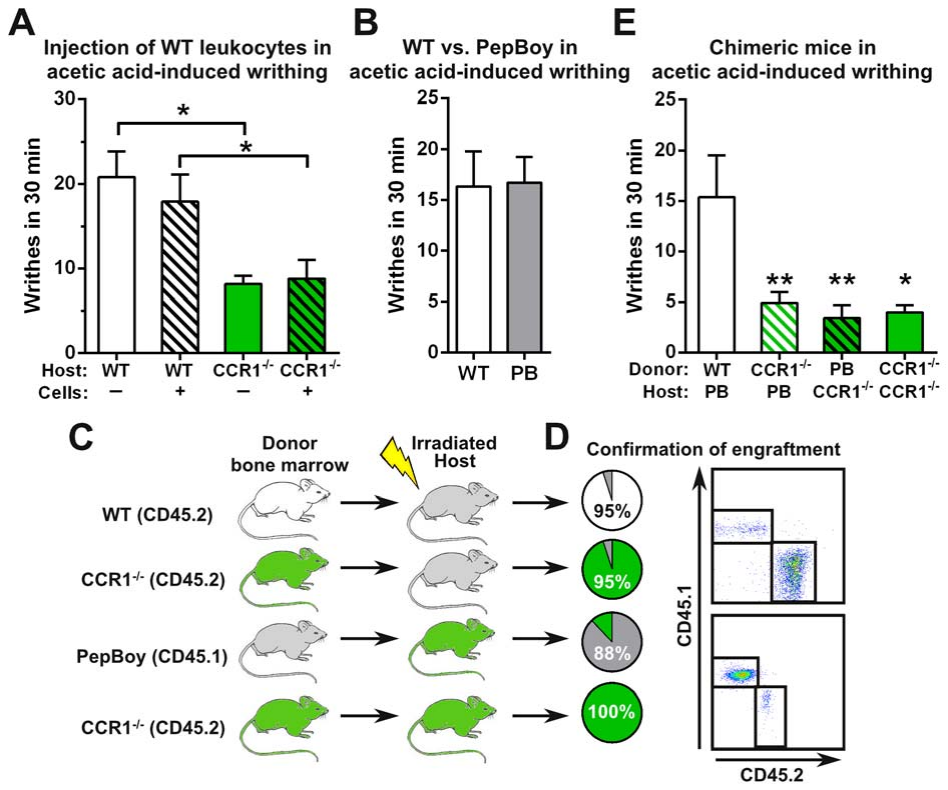
CCR1 activity on hematopoietic and non-hematopoietic cells modulates the writhing response. (*A*) WT leukocytes were isolated from the peritoneal cavity 4 hours after thioglycollate injection. 2.9 million cells were injected into WT and CCR1^−/−^ mice and immediately tested in the acetic acid-induced writhing model (*n* = 5–10). (*B*) WT and Pep Boy mice were tested in the acetic acid-induced writhing model (*n* = 10). (*C*) Bone marrow transplants were performed to generate chimeric mice using WT (CD45.2), CCR1^−/−^ (CD45.2), and Pep Boy (CD45.1) mice. (*D*) Engraftment of bone marrow was assessed after 10 weeks by measuring CD45.1 versus CD45.2 expression. (*E*) Chimeric mice were tested in the acetic acid-induced writhing model (*n* = 5–12). *P*<0.05 and **P<0.01.

We next explored whether administration of neutrophils into the inflammatory site would enhance pain responses in our acetic acid-induced writhing model. In order to obtain an enriched population of neutrophils, we used peritoneal cells isolated from mice that were treated with thioglycollate for 4 hours. Flow cytometric analysis revealed that 48% of the cells were neutrophils, 21% were B cells, and 12% were macrophages. These cells were also greater than 95% viable. To our surprise, we found that WT mice that received the injection of leukocytes responded similarly to WT mice that did not receive the cellular injection (Fig. 4A). Likewise, similar results were observed upon administration of leukocytes into CCR1^−/−^ mice. Importantly, CCR1^−/−^ mice still writhed significantly less than WT mice with or without the injection of cells. These data suggest that injection of WT leukocytes does not enhance writhing and that perhaps CCR1 activity is needed on non-immune cells.

Therefore, to more thoroughly assess the potential contribution of CCR1 on non-hematopoietic cells, we generated bone marrow chimeras utilizing B6.SJL-*Ptprc^a^ Pepc^b^*/BoyJ (Pep Boy) mice to restrict CCR1 activity to either hematopoietic or non-hematopoietic compartments. Pep Boy mice responded similarly to WT mice in our acetic acid-induced writhing model (Fig. 4B), suggesting that WT and Pep Boy mice could be used interchangeably in this model. We generated the following four types of chimeric mice to determine the role of CCR1 activity on hematopoietic and non-hematopoietic cells: 1. WT (donor) into Pep Boy (host), 2. CCR1^−/−^ into Pep Boy, 3. Pep Boy into CCR1^−/−^, and 4. CCR1^−/−^ into CCR1^−/−^ (Fig. 4C). After 10 weeks, we confirmed the successful engraftment of donor bone marrow indicated by analysis of CD45.1 and CD45.2 expression in peripheral blood and peritoneum (Fig. 4D). We next tested these chimeric mice in the acetic acid-induced writhing model. As expected, Pep Boy mice engrafted with WT bone marrow demonstrated normal signs of pain with a comparable number of writhes to non-engrafted WT mice (Fig. 4E). In contrast, Pep Boy or CCR1-deficient mice engrafted with CCR1^−/−^ or Pep Boy bone marrow cells writhed significantly less to pain induced by acetic-acid administration (Fig 4E). In fact, their inability to respond to pain was similar to chimeric mice where cells from both compartments lacked CCR1. In summary, mice with CCR1 deficiency in the hematopoietic compartment, non-hematopoietic compartment, or both all exhibited a reduced writhing response. Our results suggest that CCR1 function on both hematopoietic cells and non-hematopoietic cells must be intact in order to modulate acetic acid-induced writhing responses.

## Discussion

In this study, we confirm reports [13]–[16] that CCR1 has important roles in immune cell trafficking and inflammation and then use our CCR1^−/−^ mice and small molecule antagonists of CCR1 to uncover a novel role for CCR1 in modulating responses to pain. Through a combination of cell depletion and bone marrow reconstitution studies, we suggest that this CCR1-mediated pain response requires intact CCR1 function on both immune cells and non-hematopoietic cells. Studies with our CCR1^−/−^ mice and small molecule CCR1 antagonists show reduced immune cell trafficking in a peritonitis model and reduced disease severity in CAIA. The CCR1 ^−/−^ mice and mice treated with CCR1 antagonists also had reduced pain responses in two rodent models that coincided with reduced myeloid cell infiltration into the inflammatory site and so we explored the possibility of a direct connection between pain and immune cell activity. We depleted the dominant CCR1^+^ population, neutrophils, using antibodies, and show a reduction in pain responses. Our initial observations were consistent with reduced pain responsiveness being due solely to reduced inflammation. However, injection of neutrophils into the peritoneal cavity in CCR1^−/−^ mice did not rescue the writhing response in short term experiments, and more importantly, neither did reconstitution of the bone marrow of CCR1^−/−^ mice with CCR1^+^ hematopoietic cells (WT cells transplanted into WT hosts did have a normal writhing response). These data suggest a novel requirement for CCR1 activity on non-hematopoietic cells together with the role on immune cells for normal pain responses. These data support the rationale of CCR1 inhibition in inflammatory diseases as it could potentially reduce both inflammation and pain simultaneously through different mechanisms.

Rheumatoid arthritis is a chronic autoimmune disease characterized by migration of inflammatory cells into the synovium that leads to tissue destruction and bone degradation [17]. Current therapies that modulate the immune response have proven to be effective at reducing rheumatoid arthritis symptoms and resulting pathology; however, sustained remission is rarely achieved [18]. Severe pain is common in arthritis patients. It has been described as the most troublesome problem amongst patients with rheumatoid arthritis [19]. Additionally, pain is the area of health in which the majority of rheumatoid arthritis patients would like to see improvement [20]. This pain may be due to the inflammation or joint damage, or a combination of both. In the case of joint damage, pain can often persist even in the absence of inflammation [21]. Treatment of rheumatoid arthritis patients with a CCR1 inhibitor has been shown to reduce leukocyte numbers in synovial tissue [22]. Furthermore, recent clinical trial data demonstrate efficacy of CCR1 inhibition in rheumatoid arthritis, including reductions in pain [23]. Our results corroborate these findings and suggest that CCR1 inhibition may reduce pain, even in the absence of inflammation, due to its effects on non-hematopoietic cells.

We found that neutrophils express high levels of CCR1, traffic to inflammatory sites, and positively contribute to pain. Neutrophils are normally the first cell type to infiltrate damaged tissue and are essential for immunity and resistance to pathogens [24]. However, the role of neutrophils in pain has been debated [25], [26]. Our data demonstrating that neutrophils contribute to pain correlate well with published results showing that inhibition of the neutrophil chemoattractant leukotriene B_4_ reduced hyperalgesia and neutrophil influx into the joint in a pre-clinical arthritis model [27]. Additionally, another group found that administration of leukotriene B_4_ produced hyperalgesia that was dependent on neutrophils [11]. Neutrophils have been found to release several molecules that have hyperalgesic properties, including TNFα, IL-1β, and prostaglandins [28]. The release of hyperalgesic molecules may provide a mechanism explaining how neutrophils contribute to pain.

In the K/BxN serum-transfer mouse model of arthritis, Jacobs et al. undertook an exhaustive study to determine the role of chemokine receptors [29]. Utilizing several strains of chemokine receptor-knockout mice, these authors found that CXCR2, not CCR1, was important in their pre-clinical arthritis mouse model. This data is surprising given that CCR1 ligands were upregulated. We suspect that differences between the pre-clinical K/BxN and CAIA mouse models account for the discrepancy regarding the role of CCR1. Nevertheless, we also demonstrate that a specific CCR1 antagonist reduces arthritis in the pre-clinical CAIA mouse model. Similar results have also been shown by others [13], thus providing further confidence that CCR1 plays a major role in CAIA.

Our data suggest that CCR1 activity on non-hematopoietic cells modulates pain by an unknown mechanism. One possibility is the direct stimulation of CCR1 on sensory neurons. The inflammatory milieu contains several substances that may lead to the sensitization of sensory neurons, such as bradykinin, ATP, adenosine, cytokines, and chemokines [30]. This reduces the signaling threshold, resulting in pain to non-noxious stimuli, such as touch [31]. CCR1 has been shown to be expressed on dorsal root ganglia (DRG) neurons [32], and this is supported by our own findings (Fig. S2). There are also reports of the effects of CCR1 ligands on sensory neurons and pain. CCL3 has been demonstrated to cause neuronal calcium ion flux and enhance neuronal responsiveness to capsaicin or anandamide [33]. We also demonstrate that CCL3 and CCL5 induce calcium ion flux in neurons (Fig. S2). Likewise, CCL3^−/−^ mice have reduced pelvic pain [34]. CCL5^−/−^ mice also have reduced pain [35], and CCL5 injection into the brain results in enhanced pain [36]. Similarly, intrathecal injection of CCL7 enhanced pain, whereas administration of anti-CCL7 antibodies reduced pain. Our data demonstrating that neutrophil infiltration into the peritoneum is reduced in CCR1^−/−^ mice after acetic acid or thioglycollate injections suggest that CCR1 ligands are also elevated in these models. These data, together with our own, provide evidence that CCR1 and its ligands play an important role in pain and suggests that CCR1 ligands can act directly on sensory neurons to enhance pain. Although there is published evidence that chemokines can modulate sensory neurons and may impact pain responses, our in vivo data with CCR1^−/−^ chimeric mice removes the inflammatory contribution to pain and explicitly demonstrates that CCR1 can contribute to pain through the infiltration of neutrophils and in the absence of inflammation. Neuronal-specific knockout of CCR1 will be necessary to further elucidate the role of CCR1 on neurons in vivo.

In summary, these data suggest that CCR1 modulates pain through two independent mechanisms – neutrophil trafficking to the inflammatory site and a newly identified role on non-hematopoietic cells. As inflammatory diseases are often accompanied with infiltrating immune cells at the inflammatory site and pain, CCR1 antagonism may provide a dual benefit by restricting leukocyte trafficking and reducing pain.

## Supporting Information

Figure S1 **Anti-CCL3 and anti-CCL5 antibodies reduce acetic acid-induced writhing.** WT mice were pre-treated with anti-CCL3 and anti-CCL5 antibodies (125 µg each) or an isotype control antibody (250 µg) for two hours before the acetic acid-induced writhing was performed. Writhes were counted over the course of 30 minutes (*n* = 9–10 per group).
(TIFF)

Figure S2 **Expression and function of CCR1 on neurons.** (*A*) DRG neurons were isolated from WT and CCR1^−/−^ mice and CCR1 mRNA expression was measured by Taqman PCR (*n* = 3). Mu opioid receptor (*Oprm1*) mRNA expression was also measured as a positive control for DRG neurons (*n* = 3). ND  =  not detected. (*B*) Neurons were stimulated with increasing concentrations of CCL3 and CCL5 and calcium flux was measured (*n* = 2).
(TIFF)
