# Supplementary figures and images for: CCR1 Plays a Critical Role in Modulating Pain through Hematopoietic and Non-Hematopoietic Cells
Source: PLoS One. 2014 Aug 29;9(8):e105883. doi: 10.1371/journal.pone.0105883 (PMC4149507; doi:10.1371/journal.pone.0105883)

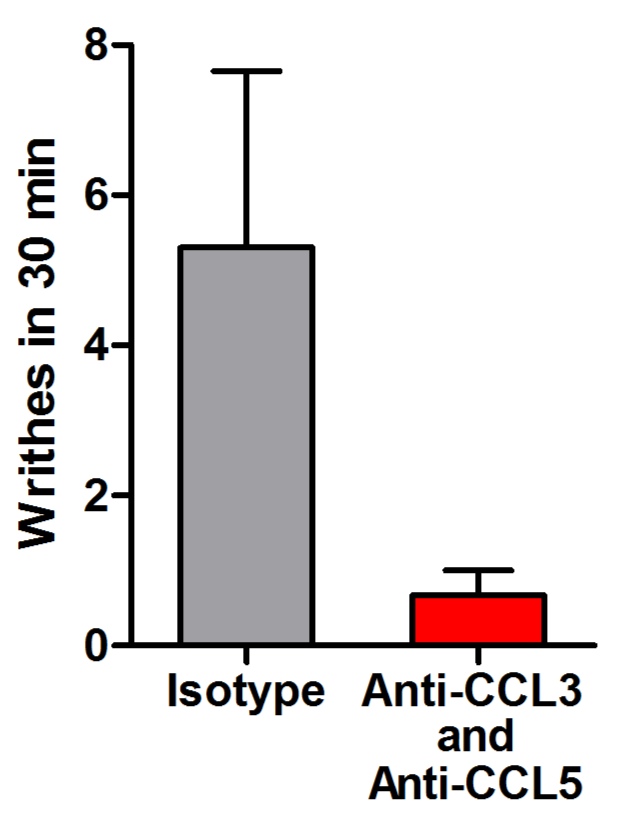

Supplement: Figure S1 — Anti-CCL3 and anti-CCL5 antibodies reduce acetic acid-induced writhing. WT mice were pre-treated with anti-CCL3 and anti-CCL5 antibodies (125 µg each) or an isotype control antibody (250 µg) for two hours before the acetic acid-induced writhing was performed. Writhes were counted over the course of 30 minutes (n = 9–10 per group). (TIFF) [file pone.0105883.s001.tiff]

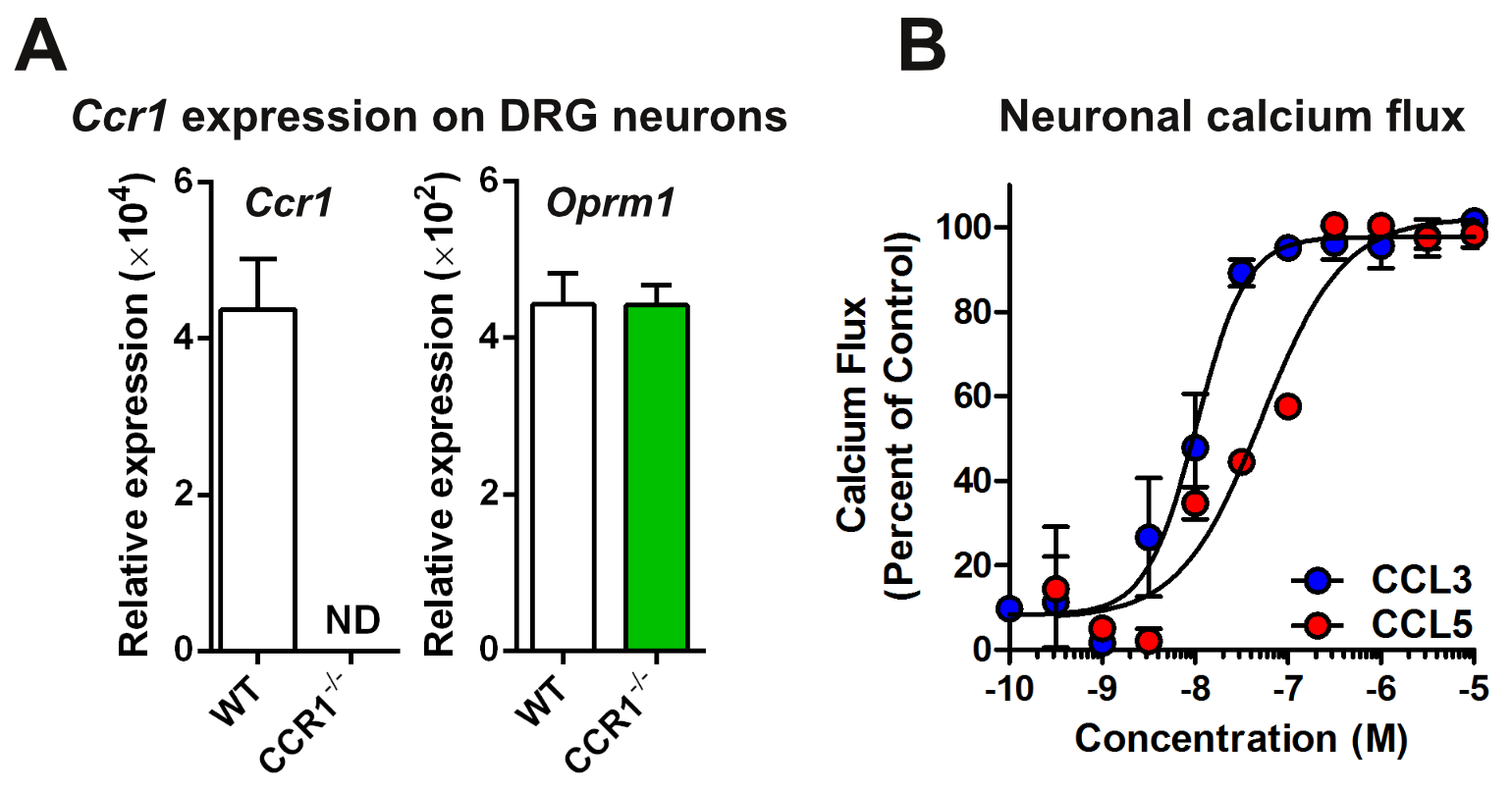

Supplement: Figure S2 — Expression and function of CCR1 on neurons. (A) DRG neurons were isolated from WT and CCR1−/− mice and CCR1 mRNA expression was measured by Taqman PCR (n = 3). Mu opioid receptor (Oprm1) mRNA expression was also measured as a positive control for DRG neurons (n = 3). ND = not detected. (B) Neurons were stimulated with increasing concentrations of CCL3 and CCL5 and calcium flux was measured (n = 2). (TIFF) [file pone.0105883.s002.tiff]
